# Differentiation of rat bone marrow mesenchymal stem cell into uterine smooth muscle cell *in vitro* study on the mechanism

**DOI:** 10.1515/biol-2025-1312

**Published:** 2026-05-25

**Authors:** ZhengYong Chen, Yuan Liao, Jing Yang, XiaoLing Zeng

**Affiliations:** Department of Obstertrics and Gynaecology, Guizhou Provincial People’s Hospital, Guiyang City, Guizhou Province, 550002, China

**Keywords:** BMSCs, USMCs, protein acetylation, miR-214, scar uterus

## Abstract

Although bone marrow mesenchymal stem cells (BMSCs) are an important resource for tissue engineering and have been shown capable of differentiating into uterine smooth muscle cells (USMCs) to aid uterine regeneration, the precise conditions and mechanisms that control this process require further investigation. This study employed a Transwell co-culture system of rat BMSCs and USMCs to investigate both the differentiation of BMSCs into USMCs and the involvement of protein acetylation and microRNA (miR)-214 in this transition. Our results showed that co-cultured BMSCs exhibited upregulated expression of smooth muscle markers (Calponin, alpha-smooth muscle actin, and smooth muscle myosin heavy chain), enhanced contractility, and decreased CD44 levels. Furthermore, we observed increased protein acetylation and a time-dependent upregulation of miR-214 during differentiation. Enhancement of either protein acetylation or miR-214 expression promoted BMSCs differentiation toward a USMC lineage, with miR-214 overexpression also elevating overall protein acetylation levels. These findings demonstrate that BMSCs can be directed to differentiate into USMC-like cells under defined *in vitro* conditions, a process likely mediated by miR-214 through promotion of protein acetylation, thereby providing mechanistic insights and experimental support for stem cell-based therapies in uterine regeneration.

## 1 Introduction

Uterine rupture during a trial of labor after cesarean delivery poses a major risk to maternal and fetal health. However, a fundamental lack of insight into the histology and molecular biology of uterine wound healing has stalled research progress [1]. The poor healing of uterine scar after cesarean section is a common complication that seriously threatens maternal health, while the quality of uterine healing after trauma can affect the pregnancy outcome of women in the subsequent second pregnancy, the formation of a scar uterus after uterine trauma, and the improvement of the quality of uterine healing after cesarean section, the risk of complications such as cesarean scar pregnancy, placenta accreta, pernicious placenta previa, uterine rupture and postpartum hemorrhage was significantly increased in the second pregnancy [2]. This represents a pressing challenge in current clinical obstetrics and gynecology.

Derived from the mesoderm, bone marrow mesenchymal stem cells (BMSCs) are defined by their multipotency, enabling them to give rise to a variety of cell types, including chondrocytes, osteoblasts, and adipocytes, when exposed to appropriate inductive signals [3], 4]. BMSCs represent an attractive and extensively investigated cell candidate for a variety of tissue engineering applications [5], 6]. BMSCs promote tissue repair both by direct differentiation and by indirectly enhancing the regenerative capacity of local stem cells through paracrine actions and immune regulation [7]. Owing to their multi-differentiation potential, paracrine activity, and immunomodulatory properties, BMSCs have emerged as a highly promising therapeutic strategy for uterine tissue regeneration and repair [8], [9], [10], [11]. This potential is particularly critical given the inherent limitations of natural healing after severe injury. While the endometrium possesses a powerful regenerative capacity, the myometrium has a limited ability to self-repair, often resulting in a prolonged healing process and incomplete recovery [12]. Therefore, a key therapeutic goal for uterine restoration is to promote the regeneration of damaged uterine smooth muscle cells (USMCs) within the myometrium, thereby re-establishing their original structure and contractile function. In rodent models of uterine injury, the transplantation of BMSCs, particularly when delivered via scaffolds, has demonstrated significant efficacy. Notably, these constructs not only enhance tissue proliferation and improve endometrial morphology but also facilitate the direct differentiation of BMSCs into endometrial stromal cells [13]. A study by Yang et al. further proposes a dual-repair model for BMSCs in myometrial regeneration: they both activate the proliferation of resident USMCs and simultaneously undergo myogenic differentiation themselves. This model is supported by *in vitro* evidence showing that BMSCs promote USMC migration and proliferation while differentiating into myogenic cells when exposed to the appropriate microenvironment [12]. However, the specific conditions and detailed molecular mechanisms governing the differentiation of BMSCs into USMCs require further elucidation.

MicroRNAs (miRNAs) are a class of highly conserved non-coding single-stranded small RNA molecules expressed by endogenous genes, which are closely related to the growth, development and cell differentiation of organisms [14]. miRNAs are critical regulators of stem cell fate, where unique miRNA signatures and mechanisms direct the differentiation of specific stem cell types into distinct functional adult cells [15]. As a well-characterized miRNA, miR-214 is located within the genomic sequence of its host long non-coding RNA, Dmn3os [16]. Research has demonstrated a context-dependent role for microRNA (miR)-214 in directing stem cell fate. While it facilitates the differentiation of embryonic stem cells into smooth muscle cells (SMCs) [17] and endothelial cells [18], it can suppress osteogenic differentiation in BMSCs [19], 20]. A consistent association with myogenic differentiation is further supported by its significant up-regulation during the differentiation of amniotic fluid stem cells into SMCs [21]. Moreover, in the context of endometrial repair, exosomal miR-214 from hypoxic endometrial epithelial cells is crucial for enhancing the migration and endometrial epithelial differentiation of mesenchymal stem cells (MSCs) [22], 23]. However, the specific function and molecular mechanism of miR-214 in the differentiation of stem cells into USMCs are still poorly understood.

Precise control of protein function is fundamental to cellular and organismal homeostasis. One such critical mechanism is acetylation, which plays a central role in the transcriptional regulation of key genes that direct the multilineage differentiation potential of MSCs [24]. The histone deacetylase inhibitor sodium butyrate (NaB) has been shown to play an important role in stem cell differentiation. NaB can promote rat BMSCs differentiation into SMCs via histone acetylation [25], 26], and plays a spatiotemporally specific key regulatory role in the differentiation of human induced pluripotent stem cells into endothelial cells, pericytes, and SMCs. The differentiation of BMSCs is critically regulated by epigenetics. While both miRNAs and histone acetylation are important epigenetic players with documented complex interplay [27], 28], their specific cooperation in directing stem cell fate remains to be fully elucidated. We therefore sought to test the specific hypothesis that histone acetylation serves as a key downstream link in miR-214-mediated phenotypic transformation of stem cells into SMCs.

The aim of this study was to investigate the differentiation of BMSCs into USMCs and the regulatory roles of protein acetylation and miR-214 within this process. To this end, we first isolated and characterized primary BMSCs and USMCs from female SD rats. We then established an *in vitro* differentiation model using a Transwell co-culture system to simulate the uterine smooth muscle niche. This setup allowed us not only to confirm the differentiation capability of BMSCs but also to delineate the function of key epigenetic and post-transcriptional regulators. We expect this work to lay the groundwork for innovative stem cell-based strategies in treating uterine trauma and preventing scar formation.

## 2 Materials and methods

### 2.1 Laboratory animals

Four-week-old female SD rats with body weight of (80–100) g were purchased from Changsha Tianqin Biotechnology Co., Ltd. (Hunan, China). Animal feeding conditions: day and night alternation 12 h light, avoid noise, single cage feeding, temperature 18–25 °C, relative humidity 45–70 %. The use of all animals was approved by the Experimental Ethics Committee of Guizhou Provincial People’s Hospital (Approval Number: No. 2024N110374), and the experiment strictly followed the consensus of author’s guidelines on Animal Ethics and Welfare published by the International Association of Veterinary Editors and the relevant local and national regulatory requirements. Minimize discomfort, pain, and death of experimental animals.

**Ethical approval:** The research related to animal use has been complied with all the relevant national regulations and institutional policies for the care and use of animals, and has been approved by the Experimental Ethics Committee of Guizhou Provincial People’s Hospital (Approval Number: No. 2024N110374).

### 2.2 Extraction and culture of BMSCs

After deep anesthesia was induced with an overdose of sodium pentobarbital (150 mg/kg, intravenous injection), the rats were euthanized by cervical dislocation. The bodies were then fully immersed in 75 % alcohol for 5 min for sterilization. Under sterile conditions, the long bones from the hind limbs were collected, and both ends of the bones were excised. The marrow cavity was repeatedly flushed with Dulbecco’s Modified Eagle Medium/Nutrient Mixture F-12 (DMEM/F-12; GIBCO, Carlsbad, California, USA, Cat. No. 11320033) to collect the bone marrow. The flushed marrow was repeatedly pipetted to achieve a homogeneous cell suspension, which was transferred to a centrifuge tube and centrifuged at 1,500 rpm for 5 min. The supernatant was discarded, and the cell pellet was resuspended in culture medium supplemented with fetal bovine serum. Cells were seeded at a density of 1 × 10^6^ cells/cm^2^ in culture flasks and placed in a 37 °C incubator with 5 % CO_2_. After 24 h, non-adherent cells were removed by washing, and cell morphology and growth were observed under a microscope. The medium was replaced every 2–3 days thereafter. When cells reached 80 % confluence, they were detached using trypsin and subcultured at a split ratio of 1: 3 or 1: 4.

### 2.3 Flow cytometry

Cell surface marker expression was assessed by flow cytometry (BD FACScalibur, BD Biosciences, San Jose, CA, USA). Cells at passage three were adjusted to a concentration of 1 × 10^6^ cells/mL. Aliquots of 1 mL cell suspension were distributed into five centrifuge tubes. According to the manufacturer’s instructions, the following antibodies () were added to the cell suspensions, which were then incubated for 1 h at 4 °C in the dark: mesenchymal stem cell (MSC)-positive markers included fluorescein isothiocyanate (FITC)-conjugated monoclonal antibody against CD29 (eBioscience, San Diego, CA, USA, Cat. No. 11-0291-82; 1 μg per test) and allophycocyanin (APC)-conjugated monoclonal antibodies against CD44 (Yeasen, Shanghai, China, Cat. No. 771567ES60; 5 μL per test), MSC-negative markers included FITC-conjugated monoclonal antibodies against CD31 (Invitrogen, Cat. No. MA5-16952; 10 μL per test) and phycoerythrin (PE)-conjugated monoclonal antibodies against CD45 (eBioscience, Cat. No. 12-0461-82; 0.25 μg per test); Corresponding isotype controls (eBioscience) were used in parallel for accurate gating. After incubation, the cells were centrifuged at 500×*g* for 5 min at 4 °C to remove unbound antibodies. The resulting cell pellets were gently vortexed and resuspended in 100 μL of staining buffer. Approximately 50,000 events were acquired for each sample, and data analysis was performed using FlowJo v10.6.2 software.

### 2.4 Extraction and culture of USMCs

After euthanasia, the rats were fully immersed in 75 % alcohol for 5 min. The uteri were then aseptically harvested. Then the uterus was transferred to a ultra-clean table and placed in a Petri dish. The uterine cavity was opened along the long axis of the uterus, and the uterine mucosa and serosal layer were scraped off. The myometrium was cut into tiny pieces and transferred into a centrifuge tube, after centrifugation, the upper layer of fluid and floating tissue were discarded. Type II collagenase solution (GIBCO, Cat. No. 17101015) was added and placed in a water bath at 37 °C for 1 h to digest. During the period, the digestion was observed. When the digestive fluid became turbid and the muscle tissue in the tube became lumpy, add serum containing medium to stop digestion, centrifugation, discard the supernatant; add the prepared serum containing high-glucose DMEM (GIBCO, Cat. No. 11965092), repeatedly blow into the cell suspension, inoculated into the flask, and cultured in the incubator. After 2 days, the fluid was changed for the first time to wash the floating tissue, and then changed every 2–3 days, during which the cell growth and morphological changes were observed, the cells grew to more than 80 % fusion and were trypsinized and passaged 1:3 or 1:4.

### 2.5 Immunofluorescence

Sterile slides were placed at the bottom of a 6-well culture plate. USMCs were seeded into the plate and cultured until they adhered to the slides. When cell confluence reached 40–60 %, the culture medium was discarded, and the cells were fixed with 4 % paraformaldehyde for 30 min. Subsequently, the cells were washed three times each with double-distilled water and phosphate-buffered saline (PBS). Permeabilization was performed using 0.3 % Triton X-100 for 10 min, followed by two washes with PBS. The cells were then blocked with 5 % bovine serum albumin for 30 min at room temperature. Primary antibodies against Calponin (Abcam, Cat. No. ab46794; 1:500 dilution), α-SMA (Abcam, Cat. No. ab124964; 1:250 dilution), and SM-MHC (Proteintech, Cat. No. 21404-1-AP; 1:250 dilution) were applied and incubated overnight at 4 °C. After washing 2–3 times with PBS, the cells were incubated with goat anti-rabbit secondary antibody (Jackson Immunoresearch Laboratories, West Grove, PA, USA, Cat. No. 111-605-003; 1:200 dilution) for 1 h at room temperature in the dark. Following another PBS wash, the cells were stained with 4′,6-diamidino-2-phenylindole (Invitrogen, Cat. No. P36931; 1 μg/mL) for 5 min at room temperature, washed 4–5 times with PBS, mounted, and finally observed under a fluorescence microscope (IX50, Olympus, Tokyo, Japan).

### 2.6 Co-culture system construction of BMSCs and USMCs

A modified transwell co-culture model of rat BMSCs and USMCs was established based on previously described methods [12]. Briefly, well-growing passage 3 USMCs were trypsinized and prepared as a cell suspension at a concentration of 2 × 10^5^ cells/mL for later use. Transwell inserts (Merck Millipore, Darmstadt, Germany, 3 μm pore size, Cat. No. CLS3414) were pre-wetted with PBS for 15 min. The inserts were then inverted over a large culture dish, and the prepared USMCs suspension was evenly seeded onto the outer surface of the membrane. An appropriate amount of culture medium was added to the dish, and it was placed in an incubator. After 6–8 h, the inserts were taken out, washed twice with PBS, flipped back to their normal orientation, and placed into a culture plate containing medium. They were returned to the incubator for another 24 h, after which the medium was refreshed. Subsequently, a prepared suspension of passage 3 BMSCs was seeded onto the inner surface of the Transwell insert (2 × 10^5^ cells/insert), which already contained USMCs on its outer surface, and co-culture was continued. The medium was replaced every 2–3 days. USMCs on the outer surface were removed at predetermined time points (0, 3, and 6 days) during the co-culture period, while induced BMSCs on the inner surface were collected for analysis of cellular phenotypes. Cells cultured under monoculture conditions (passage 3 BMSCs or USMCs) served as controls.

### 2.7 Cell treatment

To investigate the role of protein acetylation in the differentiation of BMSCs, the co-culture system of BMSCs and USMCs was treated with 3 mmol/L sodium butyrate (NaB; Sigma-Aldrich, St. Louis, MO, USA, Cat. No. B5887) for 6 days. A co-culture system without NaB supplementation served as the control. After treatment, USMCs on the outer layer of the co-culture system were discarded, while the induced BMSCs were collected for phenotypic evaluation.

### 2.8 Cell transfection

The miR-214 mimic, mimic negative control (mimic NC, Cat. No. miR1N0000001-1-10), miR-214 inhibitor, and inhibitor NC (Cat. No. miR2N0000001-1-10) were obtained from RIBOBIO biotechnology company (Guangzhou, China). Undifferentiated BMSCs were seeded in 6-well plates at a density of 2 × 10^5^ cells per well and cultured until they reached 70–90 % confluence. The cells were then serum-starved for 30 min before transfection. Transfection was performed using the Lipofectamine 3000 Kit (Thermo Fisher Scientific, Waltham, MA, USA, Cat. No. L3000015) according to the manufacturer’s protocol, with 50 nM concentrations of miR-214 mimic, miR-214 inhibitor, or their corresponding negative controls (mimic NC and inhibitor NC). Following transfection, the culture medium was replaced with fresh growth medium, and cells were harvested 72 h post-transfection for subsequent experimental analysis.

### 2.9 Reverse transcription-quantitative polymerase chain reaction (RT-qPCR)

Total RNA was extracted using Trizol reagent (Invitrogen, Cat. No. 15596018CN) and quantified with a NanoDrop 2000 spectrophotometer (Thermo Fisher Scientific). Reverse transcription of RNA into cDNA was performed using the cDNA Reverse Transcription Kit (Thermo Fisher Scientific, Cat. No. 4388950). Primer sequences were designed via the NCBI Primer-BLAST tool and commercially synthesized by Sangon Biotech (Shanghai, China); all sequences are provided in Table 1. qPCR was carried out using the SYBR Premix Ex Taq II kit (TaKaRa, Tokyo, Japan, Cat. No. DRR081A) according to the manufacturer’s instructions. The amplification protocol consisted of an initial denaturation at 95 °C for 5 min, followed by 40 cycles of 95 °C for 30 s, 57 °C for 30 s, and 72 °C for 30 s. The resulting amplification products were verified by agarose gel electrophoresis. The threshold was manually set at the lowest point of parallel log-phase amplification curves to obtain cycle threshold (CT) values for each reaction. Gene expression levels were analyzed using the 2^–ΔΔCT^ method.

**Table 1:** Primer sequences.

Gene	Forward (5′ -3′)	Reverse (5′ -3′)
α-SMA	GGG​CAT​CCA​CGA​AAC​CAC​CTA​T	CGC​CGA​TCC​AGA​CAG​AAT​ATT​TG
Calponin	CCC​ACA​ATC​ACC​ACC​ACC​ACA​AC	CCT​CGG​CCT​GAT​CTC​CCC​AAA​CT
SM-MHC	GAG​GAG​GCG​GTG​CAG​GAG​TGT​AG	GGC​GCT​GGT​GTC​CTG​CTC​CTT
CD44	AAG​ACA​TCG​ATG​CCT​CAA​AC	CTC​CAG​TAG​GCT​GTG​AAG​TG
miR-214	CCG​GAG​AGT​TGT​CAT​GTG​TC	CAG​TGC​AGG​GTC​CGA​GGT​AT
GAPDH	GGC​ACA​GTC​AAG​GCT​GAG​AAT​G	ATG​GTG​GTG​AAG​ACG​CCA​GTA
U6	GCTTCGGCAGCACATAT	TTTGCGTGTCATCCTTGC

α-SMA, alpha-smooth muscle actin; SM-MHC, smooth muscle myosin heavy chain; GAPDH, Glyceraldehyde-3-phosphate dehydrogenase.

### 2.10 Western blot

Total protein was extracted using a commercial reagent (Beyotime, Shanghai, China, Cat. No. P0013B) according to the manufacturer’s instructions. Protein concentration was determined by the bicinchoninic acid assay, and 20–40 μg of total protein from each sample was separated by sodium dodecyl sulfate–polyacrylamide gel electrophoresis (SDS-PAGE) and subsequently transferred onto a polyvinylidene fluoride (PVDF) membrane. The membranes were then blocked with 5 % bovine serum albumin for 2 h at room temperature, followed by incubation overnight at 4 °C with the following primary antibodies: Acetylated-Lysine (Cell Signaling Technology, Danvers, MA, USA, Cat. No. #9441; 1:1,000 dilution) and GAPDH (Cell Signaling Technology, Cat. No. #2118; 1:1,000 dilution). Thereafter, the membranes were incubated with horseradish peroxidase-conjugated goat anti-rabbit secondary antibody (Cell Signaling Technology, Cat. No. #7074; 1:2,000 dilution) for 1 h at room temperature. After washing three times with TBST, the blots were visualized using an enhanced chemiluminescence detection system.

### 2.11 Contractility assay

The contractility of BMSCs was measured with a carbachol challenge test, as previously described [29]. Briefly, cells were plated at 2 × 10^5^ cells per well in 6-well plates. The average percentage of cell area reduction was then calculated from images taken before and 5 min after carbachol treatment using ImageJ software, to quantify contractility.

### 2.12 Statistical analysis

Statistical analyses were performed using GraphPad Prism 10 (GraphPad Software, San Diego, CA, USA). Data are presented as mean ± standard deviation (SD). For comparisons between two groups, an unpaired *t*-test was used. For comparisons across multiple groups, one-way analysis of variance (ANOVA) was applied, followed by Tukey’s post-hoc test. A p-value of less than 0.05 was considered statistically significant.

## 3 Results

### 3.1 Identification of BMSCs and USMCs

**Figure 1:**
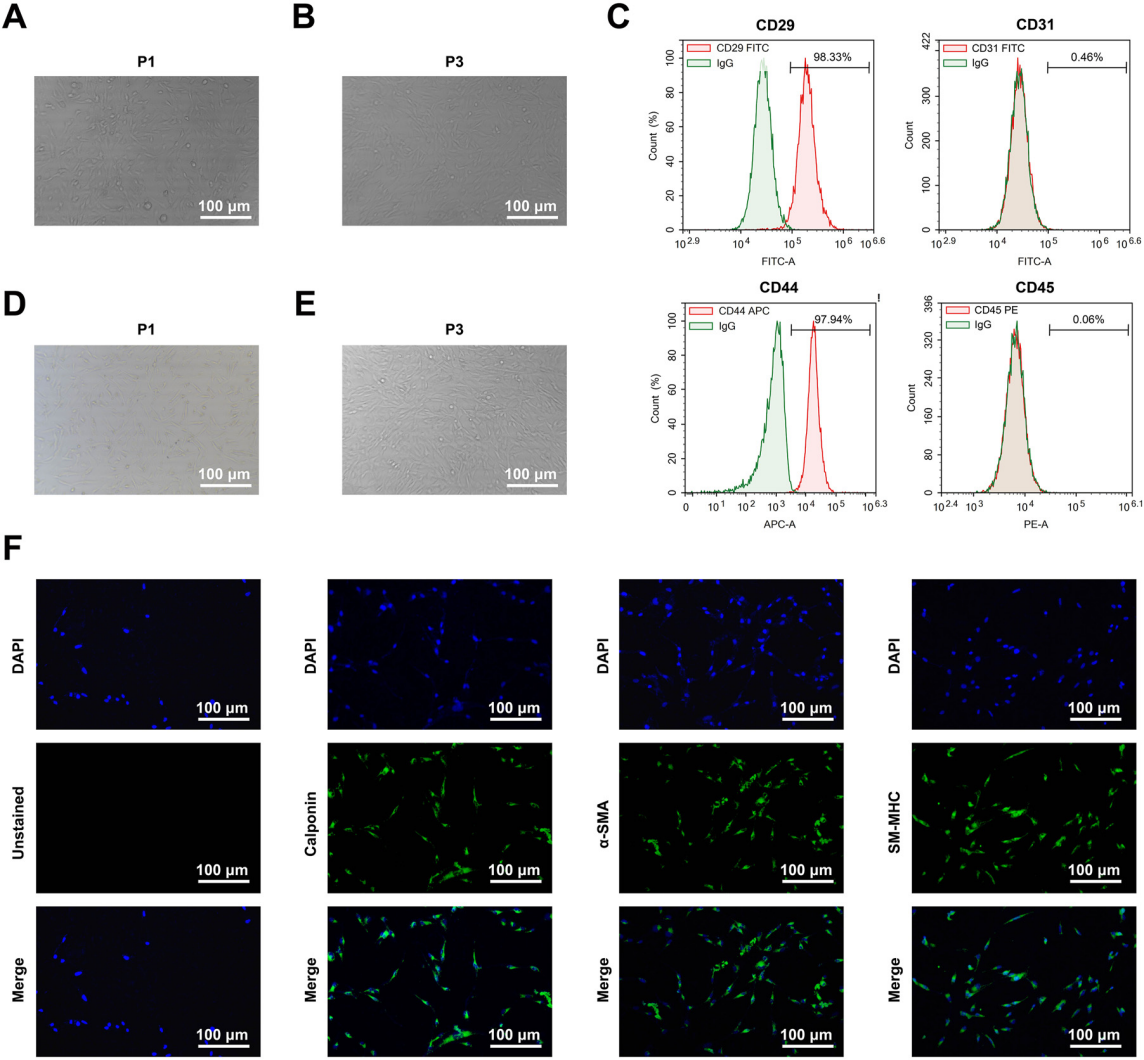
Identification of BMSCs and USMCs. Note: (A) Representative optical microscopy image of passage 1 BMSCs. (B) Representative optical microscopy image of passage 3 BMSCs. (C) Various BMSCs surface markers were analyzed by flow cytometry. (D) Representative optical microscopy image of passage 1 USMCs (E) Representative optical microscopy image of passage 3 USMCs. (F) Immunofluorescence was used to assess the expression of key USMC markers: Calponin, α-SMA, and SM-MHC. With green fluorescence indicating positivity. Scale bar = 100 μm. All experiments were performed with three independent biological replicates, and each replicate was assayed in technical triplicate.

Successful isolation of BMSCs from the rat bone marrow cavity was confirmed by morphological and immunophenotypic analyses. Primary (P1) cells showed spindle and polygonal morphology with cluster formation (Figure 1A), which evolved into a homogeneous, swirling monolayer of spindle-shaped cells by passage 3 (P3) (Figure 1B). Flow cytometry (Figure 1C) revealed high expression of mesenchymal markers CD29 (98.33 %) and CD44 (97.94 %), with negligible expression of hematopoietic/endothelial markers CD31 (0.46 %) and CD45 (0.06 %) (Figure 1C). The immunophenotypic profile confirmed that the isolated cells met the defining criteria for BMSCs, ensuring their suitability for subsequent experiments.

The isolation of USMCs was further verified. Morphological assessment under inverted phase-contrast microscopy revealed that P1 USMCs were elongated and grew in parallel or whirlpool formations (Figure 1D). At P3, the cells adopted a homogeneous spindle shape and assembled into characteristic swirling or palisading structures (Figure 1E). Furthermore, immunofluorescence results (Figure 1F) showed that over 90 % of the cells expressed the key smooth muscle markers Calponin, α-SMA, and SM-MHC. This specific immunophenotype, consistent with definitive USMC characteristics, provides a vital assurance for the ensuing research phases.

### 3.2 Co-culture drives the phenotypic transformation of BMSCs into USMCs

**Figure 2:**
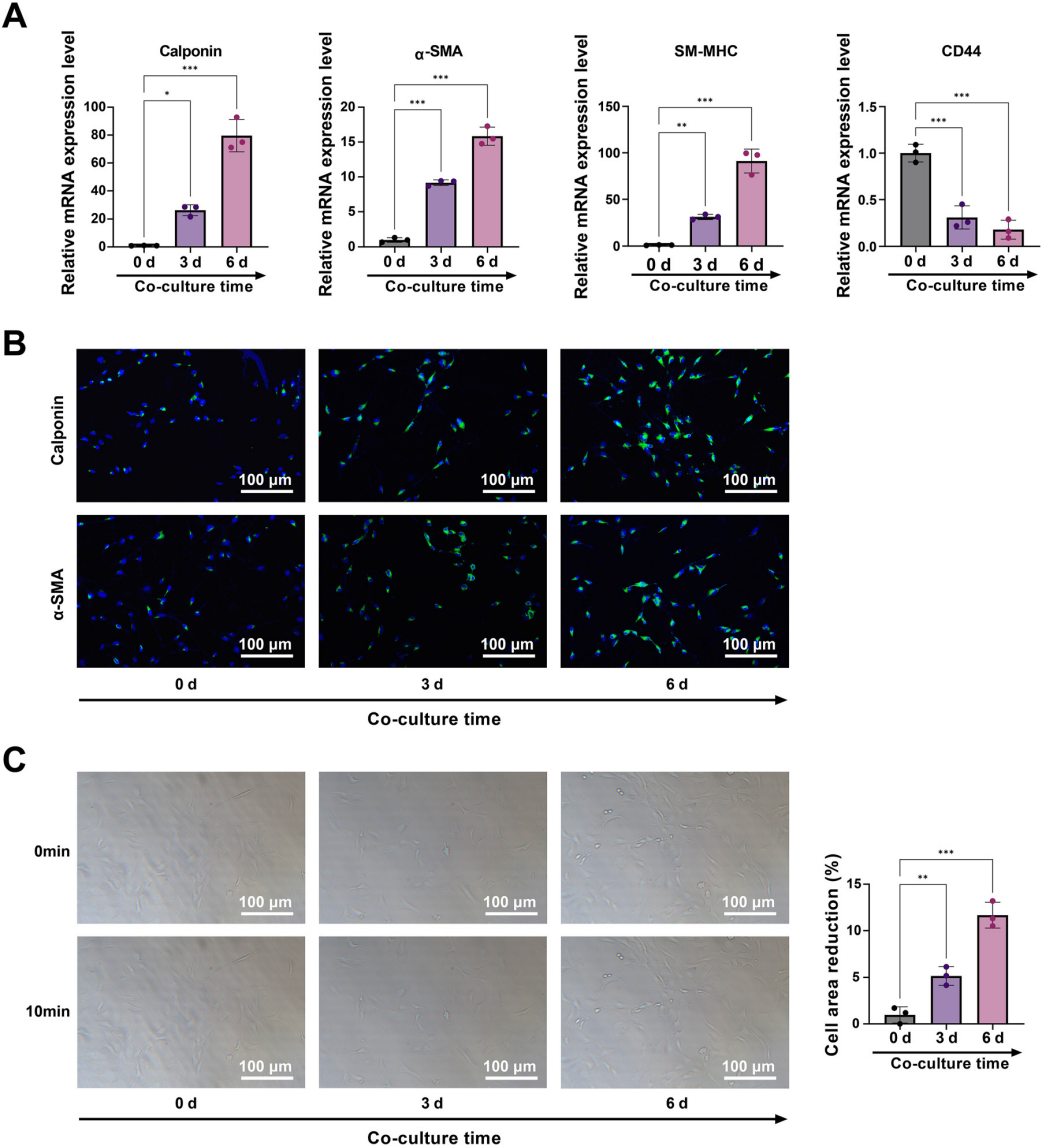
Co-culture drives the phenotypic transformation of BMSCs into USMCs. Note: (A) RT-qPCR was performed to profile the expression of Calponin, α-SMA, SM-MHC, and CD44 in BMSCs at 0, 3, and 6 days of co-culture. (B) Immunofluorescence was used to assess the expression of Calponin and α-SMA in BMSCs at 0, 3, and 6 days of co-culture. (C) Contractile response of BMSCs to carbachol stimulation following 0, 3, and 6 days of co-culture. Scale bar = 100 μm. Data are from three independent biological replicates, each assayed in technical triplicate, and presented as mean ± SD. *P < 0.05; **P < 0.01; ***P < 0.001. *P* < 0.05 was considered statistically significant.

Following a 6-day Transwell co-culture with USMCs, BMSCs adopted a smoother, muscle-like phenotype. This phenotypic shift was evidenced by a time-dependent upregulation of the smooth muscle markers Calponin, α-SMA, and SM-MHC, accompanied by the concurrent downregulation of CD44, as demonstrated by RT-qPCR and immunofluorescence results (Figure 2A and B).

Contractility assays (Figure 2C) revealed that co-cultured BMSCs contracted significantly in response to carbachol, confirming their acquisition of a contractile phenotype. Moreover, this response demonstrated a time-dependent enhancement throughout the co-culture period.

These findings collectively show that the USMC secretory microenvironment prompts the myogenic differentiation of BMSCs, and the extent of this differentiation is positively correlated with the co-culture time.

### 3.3 Protein acetylation drives the differentiation of BMSCs toward a USMC phenotype

**Figure 3:**
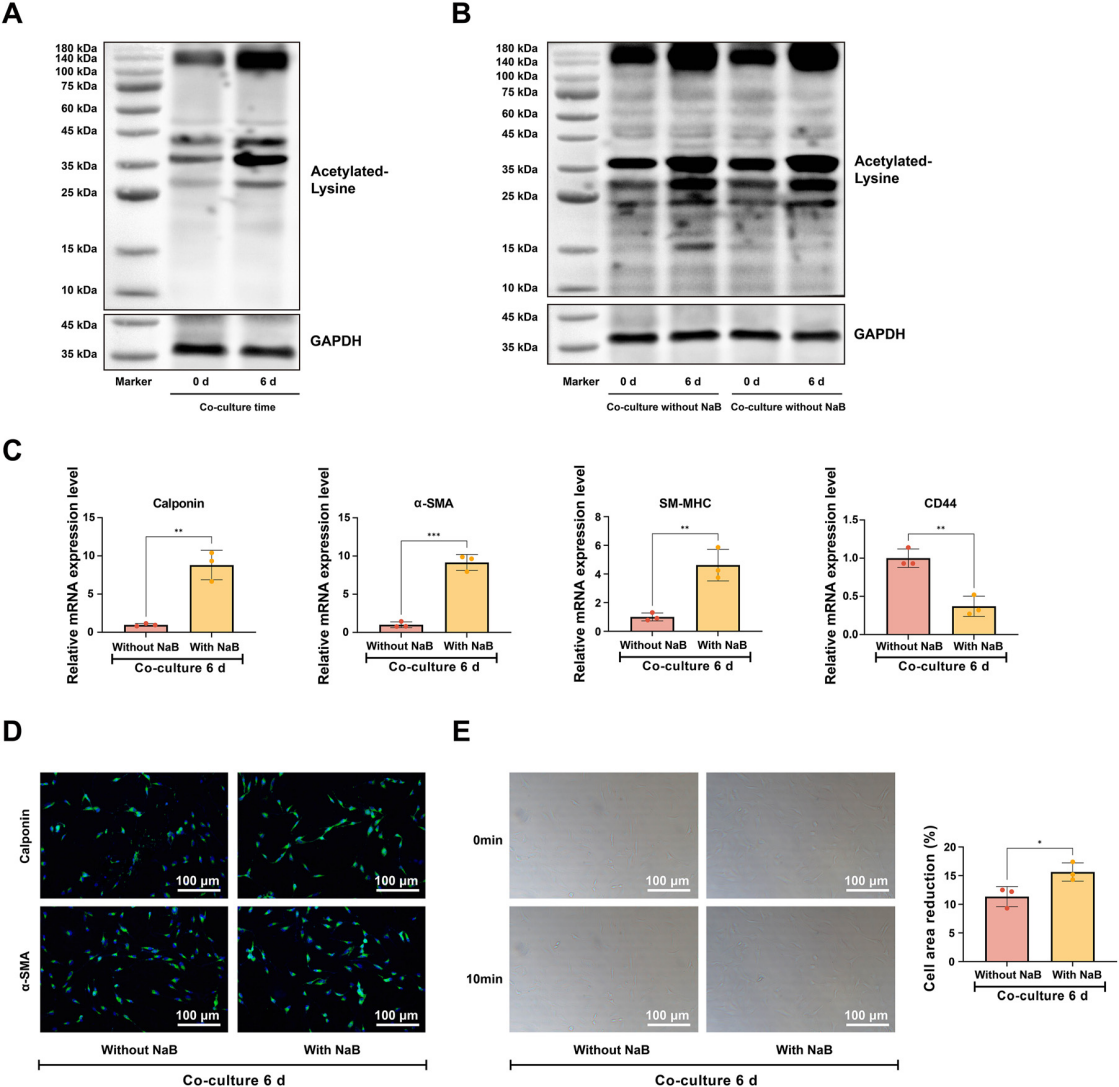
Protein acetylation drives the differentiation of BMSCs toward a USMC phenotype. Note: (A) Analysis of pan-acetylated protein levels in BMSCs by Western blot after 0 and 6 days of co-culture. (B) Analysis of pan-acetylated protein levels in BMSCs by Western blot at 0 and 6 days of co-culture in the presence or absence of NaB. (C) RT-qPCR was performed to profile the expression of Calponin, α-SMA, SM-MHC, and CD44 in BMSCs after 6 days of co-culture in the presence or absence of NaB. (D) Immunofluorescence was used to assess the expression of Calponin and α-SMA in BMSCs after 6 days of co-culture in the presence or absence of NaB. (E) Contractile response of BMSCs to carbachol stimulation following 6 days of co-culture in the presence or absence of NaB. Scale bar = 100 μm. Data are from three independent biological replicates, each assayed in technical triplicate, and presented as mean ± SD. *P < 0.05; **P < 0.01; ***P < 0.001. *P* < 0.05 was considered statistically significant.

Western Blotting analysis showed that (Figure 3A) the content of acetylated proteins in BMSCs increased significantly with the prolongation of co-culture time, suggesting that protein acetylation may be involved in the differentiation of BMSCs into USMCs. Therefore, NaB was used to observe whether it can promote the transformation of BMSCs into USMCs. Western Blotting showed (Figure 3B) that the level of protein acetylation of BMSCs in the co-culture system was significantly increased by NaB treatment compared with the control group, indicating that the addition of NaB increased the level of protein acetylation in the differentiation system.

RT-qPCR (Figure 3C) and immunofluorescence (Figure 3D) analyses confirmed that NaB treatment in the co-culture system significantly enhanced the expression of smooth muscle markers (Calponin, α-SMA, and SM-MHC) in BMSCs while reducing CD44 levels. Consistent with this molecular shift, contractility assays (Figure 3E) demonstrated that NaB-treated BMSCs exhibited a stronger contractile response to carbachol, with a significantly greater reduction in cell surface area. These results collectively demonstrate that promoting protein acetylation via deacetylase inhibition drives the functional differentiation of BMSCs into a SMC phenotype.

### 3.4 miR-214 drives BMSC differentiation into USMCs by promoting protein acetylation

**Figure 4:**
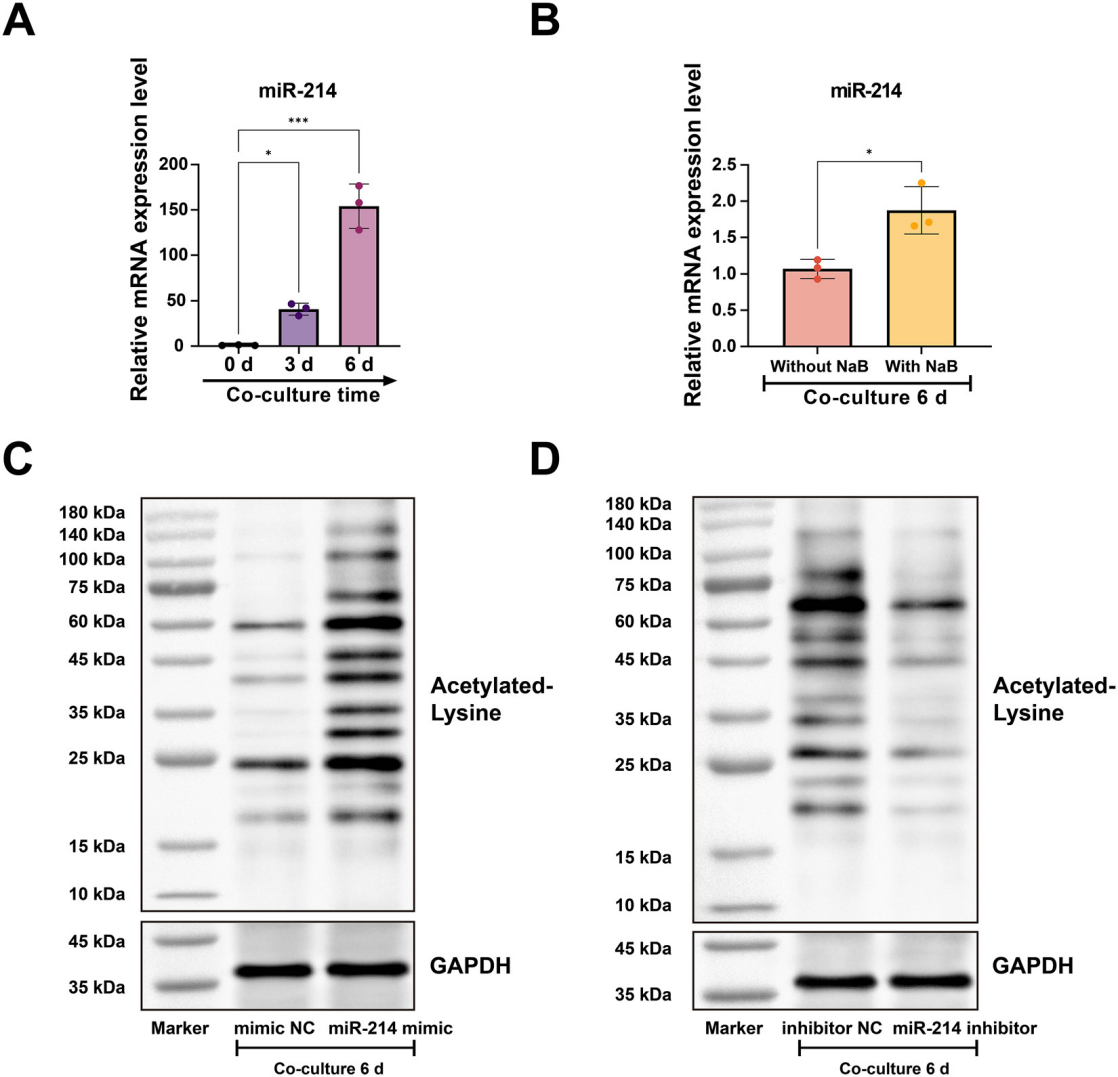
miR-214 enhances protein acetylation in co-cultured BMSCs. (A) Time-course expression of miR-214 in BMSCs during co-culture (0, 3, 6 days) measured by RT-qPCR. (B) miR-214 expression in BMSCs after 6 days of co-culture with or without NaB treatment. (C, D) Western blot analysis of pan-acetylated protein levels in BMSCs transfected with (C) mimic negative control (NC) or miR-214 mimic, and (D) inhibitor NC or miR-214 inhibitor, followed by 6-day co-culture. Scale bar = 100 μm. Data are from three independent biological replicates, each assayed in technical triplicate, and presented as mean ± SD. *P < 0.05; **P < 0.01; ***P < 0.001. *P* < 0.05 was considered statistically significant.

RT-qPCR analysis revealed a time-dependent upregulation of miR-214 expression in co-cultured BMSCs, which paralleled the induction of SMC markers, suggesting a potential role of miR-214 in driving BMSC differentiation into USMCs (Figure 4A). Notably, treatment with NaB further enhanced miR-214 expression beyond that observed in the standard co-culture system (Figure 4B), indicating a regulatory influence of protein acetylation on miR-214 levels during differentiation. To directly examine the involvement of miR-214 in protein acetylation, we modulated its expression in co-cultured BMSCs using miR-214 mimic or inhibitor. Overexpression of miR-214 significantly increased global protein acetylation, whereas its knockdown resulted in marked suppression (Figure 4C and D).

**Figure 5:**
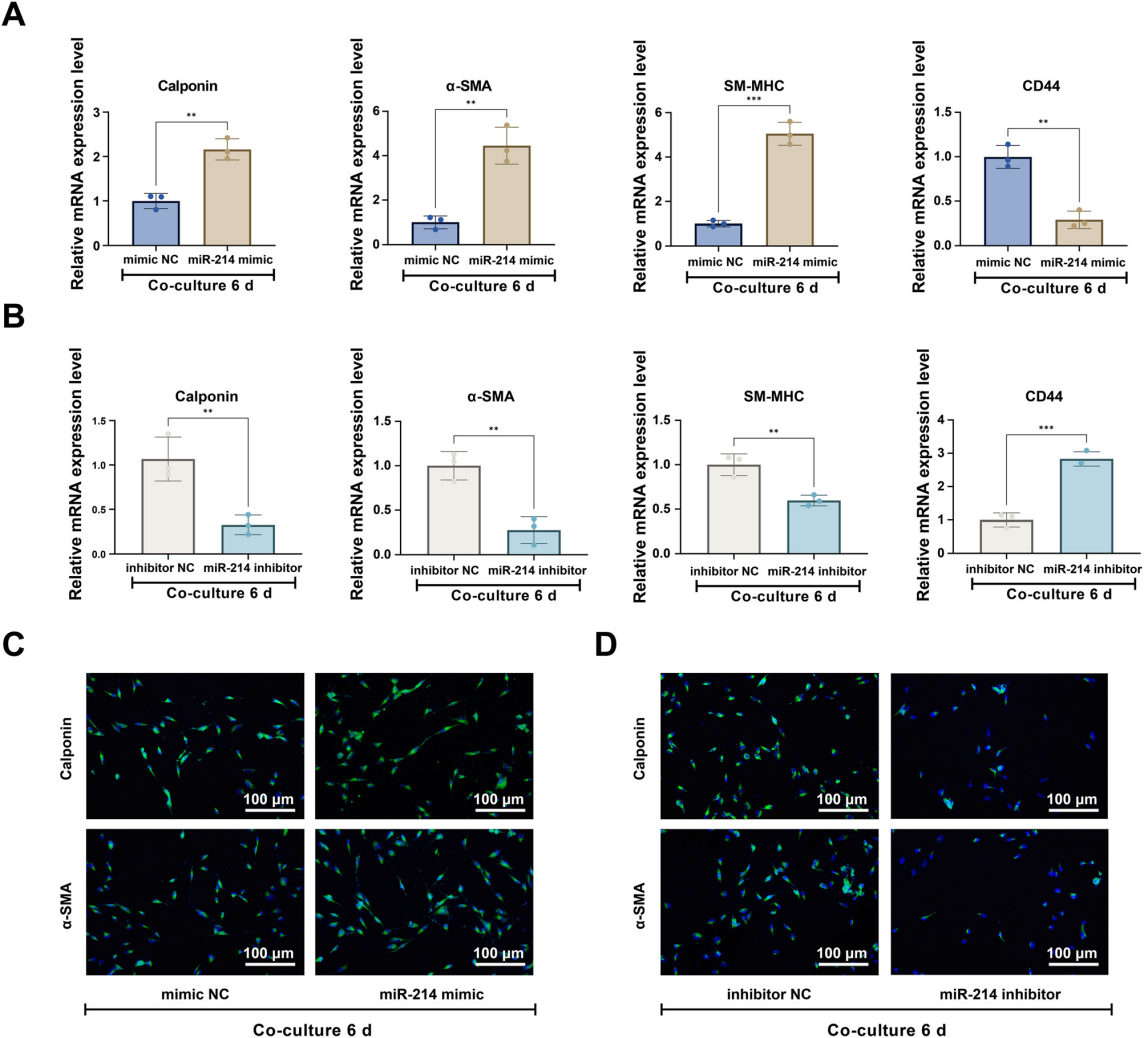
miR-214 upregulates smooth muscle marker expression in co-cultured BMSCs. (A, B) RT-qPCR analysis of Calponin, α-SMA, SM-MHC, and CD44 expression in BMSCs transfected with (A) mimic NC or miR-214 mimic, and (B) inhibitor NC or miR-214 inhibitor, after 6 days of co-culture. (C, D) Representative immunofluorescence images showing Calponin and α-SMA expression in BMSCs transfected with (C) mimic NC or miR-214 mimic, and (D) inhibitor NC or miR-214 inhibitor, following 6 days of co-culture. Scale bar = 100 μm. Data are from three independent biological replicates, each assayed in technical triplicate, and presented as mean ± SD. *P < 0.05; **P < 0.01; ***P < 0.001. *P* < 0.05 was considered statistically significant.

**Figure 6:**
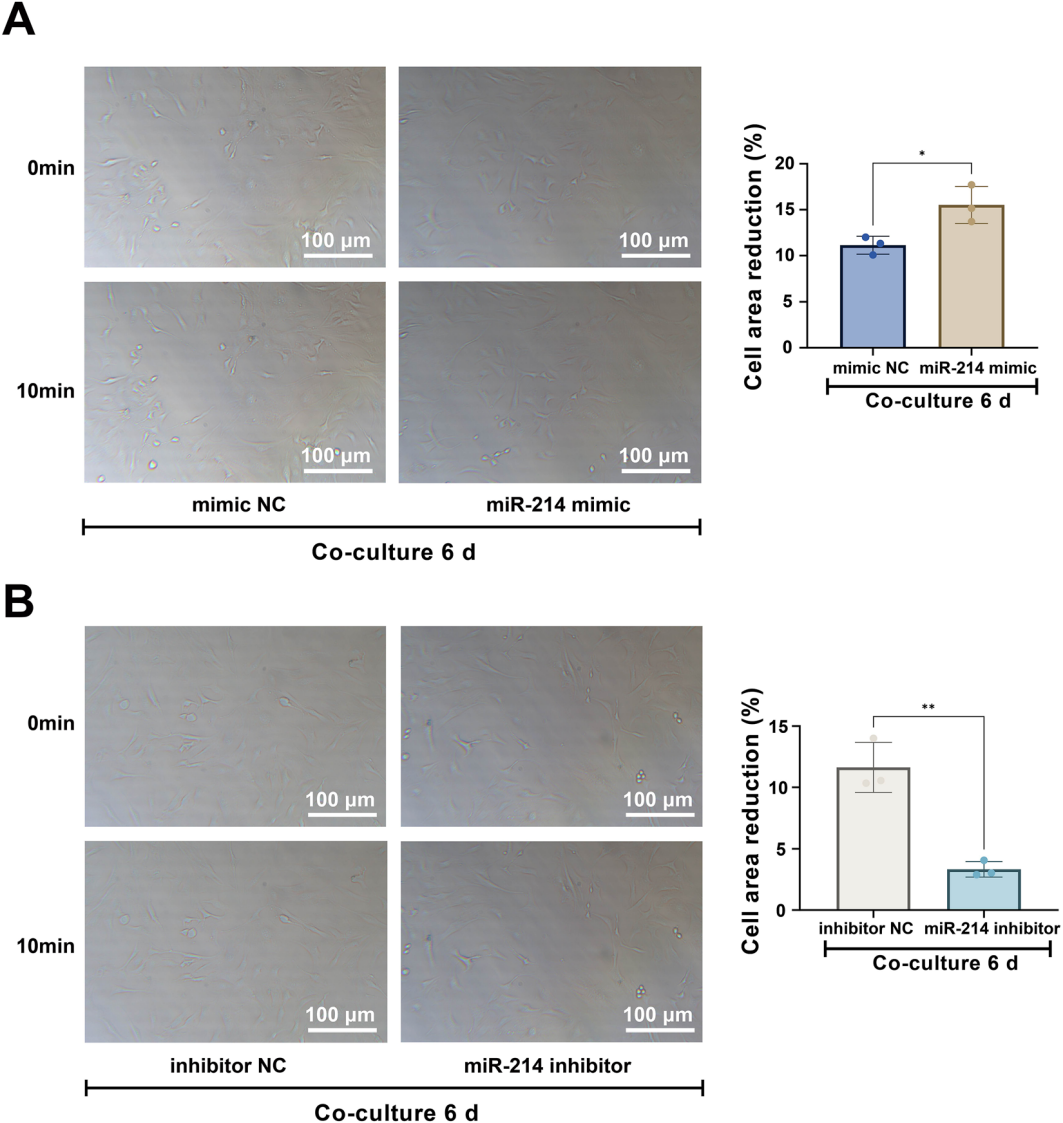
miR-214 potentiates the contractile function of differentiated BMSCs. (A, B) Contractile response to carbachol stimulation in BMSCs transfected with (A) mimic NC or miR-214 mimic, and (B) inhibitor NC or miR-214 inhibitor, after 6 days of co-culture. Scale bar = 100 μm. Data are from three independent biological replicates, each assayed in technical triplicate, and presented as mean ± SD. *P < 0.05; **P < 0.01; ***P < 0.001. *P* < 0.05 was considered statistically significant.

Further RT-qPCR (Figure 5A and B) and immunofluorescence (Figure 5C and D) analyses confirmed that upregulating miR-214 in the co-culture system markedly increased the expression of key smooth muscle markers, including Calponin, α-SMA, and SM-MHC. Conversely, inhibiting miR-214 led to a reduction in these markers. At the functional level, enhancing miR-214 expression potentiated the carbachol-induced contractile response in differentiated BMSCs, whereas miR-214 inhibition attenuated this response, consistent with the observed molecular profile (Figure 6A and B).

Collectively, these results indicate that miR-214 positively regulates the differentiation of BMSCs into USMCs, likely through a mechanism involving the promotion of protein acetylation.

## 4 Discussion

After entering a specific environment, MSCs will respond adaptively to the induced signals in the environment, and begin to transform into the cell morphology and phenotype of the surrounding cells. Current methods for inducing stem cell differentiation into target cells include cytokine induction and co-culture systems. Co-culture strategies are primarily categorized into direct contact and non-contact induction. While direct contact leverages autocrine/paracrine signaling through physical interaction, it results in cell mixing that complicates downstream analysis [30]. Non-contact induction methods, such as the Transwell system, overcome this issue by physically separating the two cell populations with a microporous membrane, enabling them to communicate via secreted factors without direct contact. Owing to this advantage, the Transwell system has been widely adopted in differentiation studies [31], 32]. To simulate the *in vivo* microenvironment for BMSC differentiation into USMCs, we employed a non-contact Transwell co-culture system. BMSCs and USMCs were seeded in the upper and lower chambers, respectively. A membrane pore size of 1.0 µm was selected to permit the exchange of soluble signaling factors and maintain gap junction communication, yet prevent cell migration. This setup successfully promoted the myogenic differentiation of BMSCs, corroborating earlier reports [12].

Histone acetylation is co-regulated by histone acetyltransferases (HATs) and histone deacetylases (HDACs). HATs activate gene expression by adding acetyl groups to the lysine-rich amino-terminal tails of histones [33], 34]. In contrast, HDACs function by catalyzing the deacetylation of histone lysine residues. This process maintains the positive charge on lysine residues, leading to tighter binding between histones and DNA, thereby suppressing gene transcription [35]. HDACs are divided into four major classes (I, II, III, and IV), which differ in structure, subcellular localization, and cofactor requirements [36]. Among them, Class II HDACs (HDAC4/5/7/9) interact with the serum response factor (SRF) and myocyte enhancer factor 2. They also suppress aberrant SMC gene expression through the ability of SRF to bind to the CArG-box DNA sequence and to Myocardin in chromatin [37]. As one of the most extensively studied HDACs inhibitors, NaB is a short-chain fatty acid produced by gut microbiota. Its primary action is the broad inhibition of Class I and II HDACs. NaB acts by competitively binding to zinc ions in the catalytic domain of HDACs and influences cellular processes such as proliferation, differentiation, and gene expression by modulating histone deacetylation [38]. Previous studies have shown that NaB can regulate the differentiation of stem cells into various lineages [26]. Our study observed the occurrence of protein acetylation during the differentiation of stem cells into SMCs. Importantly, NaB enhanced the phenotypic transformation of BMSCs into USMCs by increasing histone acetylation levels. Research by Liu et al. [25] demonstrated that NaB effectively promotes the differentiation of BMSCs into SMCs. NaB significantly suppressed the expression and enrichment of HDAC2 at SMC-specific genes in MSCs, which subsequently induced elevated acetylation of H3K9 and H4, leading to the expression of SMC marker genes such as α-SMA, Calponin, and SM-MHC. Similarly, our findings revealed a significant upregulation of α-SMA, Calponin, and SM-MHC following NaB treatment. Therefore, the core mechanism by which NaB promotes the phenotypic transformation of BMSCs into USMCs is likely through the inhibition of HDACs, which elevates genome-wide histone acetylation levels, resulting in a more open chromatin conformation. This enhances acetylation at the promoter regions of SMC-specific genes such as α-SMA, Calponin, and SM-MHC (e.g., H3K9ac and H4 acetylation), making these genes more accessible to transcription factors such as SRF, thereby initiating differentiation. Although NaB may primarily initiate the differentiation process through broad HDAC inhibition, the specific cell fate commitment, such as toward a uterine smooth muscle lineage, is likely determined by the combined actions of more specific epigenetic regulators and transcription factors. For instance, in our study, differentiation took place under co-culture conditions with USMCs. This implies that cytokines, growth factors, and other signals secreted by USMCs provide lineage-specific instructions to BMSCs. The “open” chromatin environment fostered by NaB enables stem cells to respond more efficiently to these specific signals. Thus, the precise upstream and downstream mechanisms through which NaB promotes differentiation warrant further in-depth investigation.

miRNAs are potent genetic regulators capable of orchestrating entire cellular pathways by targeting multiple genes simultaneously. This broad regulatory capacity consequently makes them attractive therapeutic candidates for correcting dysfunctional cellular processes in various diseases [39]. Their critical role in stem cell differentiation and myogenesis is well-established [40], 41]. Notably, miR-214 has been identified as a key facilitator of stem cell differentiation into SMCs [17]. We observed a concurrent, time-dependent increase in the expression of miR-214 and SMC markers during co-culture. Crucially, overexpression of miR-214 was found to promote the differentiation of BMSCs into USMCs by enhancing protein acetylation. miRNAs are known to regulate gene expression post-transcriptionally through imperfect complementarity with the 3′ untranslated region of their target mRNAs. This interaction leads to either mRNA decay or translational inhibition, ultimately controlling protein output [42]. Thus, the functional role of miR-214 in this differentiation process is likely mediated by its regulation of protein acetylation through specific target genes. According to target prediction from the bioinformatics database ENCORI (https://rnasysu.com/encori/index.php), miR-214 can target several HDACs, including HDAC5 and HDAC9. Furthermore, studies have reported that miR-214 can regulate cellular processes in various diseases by targeting SIRT1 and SIRT3 [43], [44], [45], both of which belong to the class III HDACs. This collective evidence suggests a potential mechanism through which miR-214 modulates protein acetylation. Although the identification of its direct targets was not feasible within the scope of the present study, we believe this direction holds significant value for the field, and a detailed investigation of this mechanism is a critical objective for our ongoing and future work.

This study has several limitations. First, the differentiation of BMSCs into USMCs was verified only within a specific *in vitro* microenvironment. While this model provides a valuable platform for mechanistic investigation, it must be acknowledged that cell culture systems cannot fully replicate the complex physiological and biomechanical microenvironment present during uterine tissue repair, which includes critical processes such as vascularization, immune cell infiltration, and dynamic remodeling of the extracellular matrix. Consequently, the extrapolation of our findings to *in vivo* physiological or pathological conditions must be approached with caution. Existing literature on uterine repair highlights that the recruitment and functional integration of stem cells within the injured endometrium are finely regulated by multiple *in vivo* factors [46], 47], which may differ substantially from our *in vitro* observations. For instance, Huang et al. demonstrated that human amniotic MSCs promoted endometrial repair primarily through paracrine mechanisms rather than transdifferentiation [48]. Therefore, the incorporation of an *in vivo* uterine injury or regeneration model would significantly enhance the clinical relevance and potential application of our findings. Second, the mechanistic insights presented here are preliminary. Future studies will focus on systematically identifying and validating the network of genes directly targeted by miR-214 under conditions that mimic the uterine microenvironment. Such work is essential to fully elucidate the molecular pathway by which miR-214, via epigenetic regulation, directs the differentiation of BMSCs toward a USMC lineage, thereby providing a more solid theoretical foundation for stem cell-based tissue engineering strategies in uterine repair.

## 5 Conclusions

Our study provides evidence that BMSCs possess the capacity to differentiate into a USMC lineage under defined *in vitro* conditions. This differentiation is driven, at least partially, by miR-214 through its role in promoting protein acetylation. These findings enhance our mechanistic understanding of stem cell differentiation and contribute an experimental basis for potential future applications in uterine trauma repair and regenerative medicine.
